# The health status burden of people with fibromyalgia: a review of studies that assessed health status with the SF-36 or the SF-12

**DOI:** 10.1111/j.1742-1241.2007.01638.x

**Published:** 2008-01

**Authors:** D L Hoffman, E M Dukes

**Affiliations:** 1 New Haven CT, USA; 2 Pfizer, Inc. New York, NY, USA

## Abstract

**Objective:**

The current review describes how the health status profile of people with fibromyalgia (FM) compares to that of people in the general population and patients with other health conditions.

**Methods:**

A review of 37 studies of FM that measured health status with the 36-item Medical Outcomes Study Short-Form Health Survey (SF-36) or the 12-item Short-Form Health Survey (SF-12).

**Results:**

Studies performed worldwide showed that FM groups were significantly more impaired than people in the general population on all eight health status domains assessed. These domains include physical functioning, role functioning difficulties caused by physical problems, bodily pain, general health, vitality (energy vs. fatigue), social functioning, role functioning difficulties caused by emotional problems and mental health. FM groups had mental health summary scores that fell 1 standard deviation (SD) below the general population mean, and physical health summary scores that fell 2 SD below the general population mean. FM groups also had a poorer overall health status compared to those with other specific pain conditions. FM groups had similar or significantly lower (poorer) physical and mental health status scores compared to those with rheumatoid arthritis, osteoarthritis, osteoporosis, systemic lupus erythematosus, myofacial pain syndrome, primary Sjögren's syndrome and others. FM groups scored significantly lower than the pain condition groups mentioned above on domains of bodily pain and vitality. Health status impairments in pain and vitality are consistent with core features of FM.

**Conclusions:**

People with FM had an overall health status burden that was greater in magnitude compared to people with other specific pain conditions that are widely accepted as impairing.

**Review Criteria**

Studies in this review were identified through a search of electronic databases (MEDLINE: 1990–2006; EMBASE: 1990–2006). Search terms included: ‘fibromyalgia’, ‘health status’, ‘quality of life’, ‘SF-36’ and ‘SF-12’. Reference lists from published articles were also searched. Studies were selected if they were published in the English language between 1990 and (March) 2006 and assessed health status with a validated version of the SF-36 or the SF-12.

**Message for the Clinic**

Although FM is a controversial construct, studies performed worldwide showed that the health status profile of people with FM was remarkably consistent. People with FM had significant impairments in both mental and physical health status domains. People with FM had a poorer overall health status than people with specific pain conditions that are widely accepted as impairing.

## Introduction

Fibromyalgia (FM) is a chronic pain condition that is estimated to affect 2–3% of the general population (1–3). FM is nine times more prevalent in women than in men (4). FM is characterised by chronic widespread pain. Common problems related to FM include fatigue, sleep disturbance, morning stiffness, paraesthesias, headache and concurrent medical and psychiatric disorders (5,6). The cause of FM pain is not known, although it is generally agreed that patients with FM (5) have a dysregulation of central sensory processing frequently referred to as ‘central centralisation’ (6–8). The presence and severity of FM cannot be determined by objective clinical findings, radiographic abnormalities or routinely used laboratory tests (9). Presently, there is no known cure for FM. Treatment of FM is focussed on alleviating pain and increasing function.

In 1990, the ACR published criteria to classify FM for research (5); these criteria require the presence of chronic widespread pain in combination with tenderness on examination at 11 or more of 18 anatomical sites known as tender points. Publication of the ACR criteria heralded a dramatic increase in FM research (6), including studies quantifying the health status impact of the condition. This review synthesises information on the health status burden of FM. We focussed on studies that measured health status with the 36-item Medical Outcomes Study (MOS) Short-Form Health Survey (SF-36) (10) or the abbreviated 12-item Short-Form Health Survey (SF-12) (11). The SF-36 and the SF-12 measure the same concepts of physical, mental and social functioning. The SF-36 and the SF-12 are generic health status instruments as opposed to instruments that target a particular disease. Both instruments permit comparisons across groups with different health conditions and they have been widely applied in studies worldwide.

It is important to understand the health status burden of people with FM. Health status data quantify impairments in physical, mental and social functioning. Such information can highlight areas where people with FM experience particular difficulty and where healthcare providers may be able to effect change in clinical status. Data on the relative health status impact of different health conditions can also be used to help inform healthcare policy. In particular, this review focuses on how the health status profile of people with FM compares to that of people with painful conditions, such as rheumatoid arthritis (RA), osteoarthritis (OA), systemic lupus erythematosus (SLE), headache and others. FM is a controversial construct. If studies from around the world were to reveal a consistent and serious pattern of impairment among people with FM, findings would stress the importance of addressing the health status burden of FM, irrespective of debate about how FM should be classified.

This review addresses four questions about the health status burden among people with FM. Which health status domains appear to be most affected in people with FM? How does the health status burden in people with FM compare to that of people in the general population and patients with other specific health conditions? Do health status profiles differ between male and female patients with FM? To what extent does FM contribute to the overall health status burden among people who have another specific pain condition at the same time (i.e. a concurrent condition)?

## Methods

### Literature search

Studies in the current review were identified through searches of electronic databases (MEDLINE from 1990 to 2006; EMBASE from 1990 to 2006). Search terms included ‘fibromyalgia’, ‘SF-36’, ‘SF-12’, ‘health status’ and ‘quality of life’. The search strategy included the term ‘quality of life’ because the SF-36 and the SF-12 are concurrently classified as measures of health status and health-related quality of life. Reference lists of published articles were also manually searched.

### Selection criteria

We selected studies that examined the health status burden of people with FM using a validated version of the SF-36 or the SF-12. Studies were included if they were published in peer-reviewed journals in the English language between 1990 and (March) 2006.

### Classification of diagnostic groups

We did not limit the review to studies that used published classification criteria for FM or any other pain condition, as classification criteria developed for research purposes may not be applied in routine clinical practice. Of the 37 studies that met criteria for inclusion in this review, 32 (86%) classified FM according to 1990 ACR criteria (5). Of the five remaining studies, two (12,13) relied on a physician's clinical diagnosis; one (14) classified survey respondents according to whether they reported having received a diagnosis of FM and/or one of the other pain conditions under study; one (15) did not report on the system used to classify FM or the other health conditions under study; and, one (16) defined FM using the 1990 ACR criteria for widespread pain but not on the presence of tender points [which was the same definition used by two (17,18) other studies to define ‘chronic widespread pain’ (CWP)]. Of the 14 studies that also examined another pain condition, 11 (79%) used published criteria (19–26) to classify participants into the respective diagnostic group.

### Health status measures

All studies reviewed measured health status with the SF-36 (*n* = 34) or the SF-12 (*n* = 3). The SF-36 and SF-12 have been validated in multiple languages (10,11,18,27–31). The SF-36 produces eight scale scores for eight domains of health status: physical functioning, role functioning difficulties caused by physical problems, bodily pain, general health, vitality, social functioning, role functioning difficulties caused by emotional problems and mental health. Scale scores range from 0 to 100, with higher scores indicating better functioning. Table 1 shows SF-36 normative data for MOS patients with five different health conditions: hypertension, recent acute myocardial infarction (MI), type II diabetes, chronic obstructive pulmonary disorder (COPD) and clinical depression. Comparing FM scores to the published norms can help place the health status of FM into context.

**Table 1:** Normative data for the SF-36

		Mean SF-36 Scale Scores (SD)							
		
	n	PF	RP	BP	GH	VT	SF	RE	MH
**General population**									
US general population in 1998 (10)	2474	84.2 (23.3)	81.0 (34.0)	75.2 (23.4)	72.0 (20.3)	60.9 (21.0)	83.3 (23.0)	81.3 (33.0)	74.7 (18.1)
US general population in 1998,women age 45–54* (10)	193	82.9 (21.7)	80.0 (35.4)	72.1 (23.3)	70.5 (20.6)	60.6 (21.3)	82.7 (20.8)	81.9 (33.3)	74.4 (18.1)
Dutch general population in 1998† (14)	3664	82.5 (24.8)	77.7 (37.8)	80.2 (23.6)	69.4 (19.6)	65.9 (20.0)	84.2 (23.1)	87.2 (30.6)	77.3 (17.1)
**MOS patient norms (10)**									
Hypertension	2089	73.4	62.0	72.3	63.3	58.3	86.7	76.7	77.9
Recent acute MI	107	69.7	51.4	72.6	59.2	57.7	84.6	73.5	75.8
COPD	85	56.9	34.4	54.8	45.3	44	71.8	59.7	68.1
Congestive heart failure	216	47.5	34.4	62.7	47.1	44.3	71.3	63.7	74.7
Type II diabetes	541	67.8	56.6	68.5	56.1	55.7	82.0	75.6	76.74
Clinical depression	502	71.6	44.4	58.8	52.9	40.1	57.2	38.9	46.3

SF-36, short-form health survey (36 item): PF, physical functioning; RP, role functioning difficulties caused by physical problems; BP, bodily pain; GH, general health; VT, vitality; SF, social functioning; RE, role functioning difficulties caused by emotional problems; MH, mental health. Higher scores indicate better health status. Patient norms: MOS, Medical Outcomes Study; MI, myocardial infarction; COPD, chronic obstructive pulmonary disorder; HTN/OA, comorbid hypertension and osteoarthritis.

* Normative data are presented for this demographic group because FM study groups typically comprised female patients whose average age fell within the range of 45–54 years.

† Weighted for the Dutch age-sex population.

SF-36 scale scores can be used to derive two summary measures of health status: physical component summary (PCS) and mental component summary (MCS). The PCS includes scales assessing physical functioning, role functioning difficulties caused by physical problems, bodily pain and general health. The MCS includes scales assessing vitality, social functioning, role functioning difficulties caused by emotional problems and mental health. The PCS and MCS are standardised to reflect a general population mean of 50 and a SD of 10. Higher scores represent better functioning.

The SF-12 was derived from the SF-36 (11). The SF-12 measures the same health status concepts as the SF-36. However, the SF-12 version 1 (11) (the only version studies used in this review) only yields scores for the physical (PCS) and mental (MCS) summary measures. The description of the SF-12 PCS and MCS scores is the same as that described for the SF-36 PCS and MCS scores described above.

### Presentation of findings

Findings are presented in four sections. Section 1 describes findings from general population surveys. General population surveys collect data from people randomly selected from the community. An advantage of general population surveys is that findings are not biased by help-seeking. The term ‘respondent’ is used when describing findings from general population surveys. Section 2 describes findings from cross-sectional studies that recruited patients from outpatient medical settings. Section 3 describes findings from FM clinical trials. FM clinical trials are the only longitudinal studies in this review. Health status scores are described for the total study sample at baseline. Discussion of treatment outcomes is limited to the number of trials that found a significant change from baseline on one or more aspects of health status. It is beyond the scope of this review to provide a detailed discussion of treatment effects associated with different therapies. Section 4 describes the extent to which impairments of people with FM are because of the presence of FM itself as opposed to the presence of a concurrent pain condition. Findings help to show the extent to which FM contributes to the overall health status burden in afflicted persons. In each section, tables present mean SF-36 or SF-12 scores, when numeric score values were reported in the original publications. Differences based on a statistical analysis with p-values of < 0.05 are described in the text.

### Statistical analysis

This review presents findings as reported in the original publications, with two exceptions where we performed our own analysis on the published data. In section 1, published SF-36 data from a Dutch general population study (14) were used to test for differences between the FM group and each of the other 11 pain condition groups under study. The difference between the two groups was considered to be significant at the 0.05 level, it was larger than 1.96 times the square root of the sum of the squared standard errors of both groups (14, p. 724). In section 3, when baseline health status data were presented separately for treatment groups rather than for the total clinical trial sample, we calculated mean values for the total sample by averaging scores across treatment groups.

## Results

### Section 1: general population surveys

The SF-36 was used to examine the health status of people with FM and various other pain conditions in the general populations of the Netherlands (14; Table 2) and Sweden (17) (data not shown). People in each of the pain condition groups had significantly lower (poorer) mean scores on all eight health status domains compared with people in the general population (14,17). Health status impairments among people with FM were especially pronounced. People with FM had similar, and in most cases significantly lower, health status scores compared with those in various other pain condition groups (Table 2). Study groups with FM and CWP also scored significantly lower than the group with chronic regional pain on all eight health status domains (17). Findings highlight that people with FM in the general population have a poorer overall health status than those with widely accepted pain conditions, including RA, OA and osteoporosis.

**Table 2:** SF-36 scores among persons in the general population of the Netherlands, by pain condition (14, p. 725)

		Mean SF-36 Scale Scores (SE)							
		
Pain condition group	n	PF	RP	BP	GH	VT	SF	RE	MH
FM	43	55.0 (3.2)	41.4 (5.8)	48.2 (3.6)	50.1 (3.0)	39.9 (3.1)	60.3 (3.4)	81.5 (4.8)	64.1 (2.6)
Herniated disk	368	**73.2** (1.1)	**65.8** (2.0)	**67.3** (1.3)	**62.9** (1.1)	**61.4** (1.1)	**77.7** (1.2)	82.6 (1.7)	**73.2** (0.9)
Gout	138	**75.6** (2.0)	**68.1** (3.6)	**70.2** (2.2)	**64.7** (1.9)	**60.8** (1.9)	**79.1** (2.2)	78.7 (3.0)	**73.2** (1.7)
Repetitive strain injury	63	**73.5** (2.5)	**65.1** (4.4)	**64.5** (2.7)	**64.9** (2.3)	**60.2** (2.4)	**79.2** (2.7)	82.7 (3.7)	**72.8** (2.0)
Epicondylitis	418	**80.5** (1.1)	**68.1** (1.9)	**71.0** (1.2)	**67.8** (1.0)	**63.1** (1.0)	**82.4** (1.1)	82.8 (1.6)	**75.1** (0.9)
OA of knee	547	**67.6** (1.0)	**61.0** (1.9)	**62.7** (1.1)	**60.1** (1.0)	**58.8** (1.0)	**75.7** (1.1)	80.4 (1.6)	**72.0** (0.9)
OA of hip	354	**62.4** (1.4)	**52.8** (2.5)	**59.1** (1.5)	**60.0** (1.3)	**56.8** (1.3)	**73.2** (1.5)	80.5 (2.1)	**73.5** (1.2)
Osteoporosis	280	**64.3** (1.4)	**55.9** (2.6)	**60.9** (1.6)	**58.6** (1.3)	**56.7** (1.4)	**69.8** (1.6)	77.2 (2.2)	68.9 (1.2)
Whiplash	79	**72.3** (2.3)	**57.6** (4.2)	**62.7** (2.6)	**63.0** (2.2)	**58.3** (2.3)	**77.3** (2.5)	78.0 (3.5)	**72.3** (1.9)
RA	156	62.3 (2.0)	49.0 (3.5)	**58.0** (2.2)	52.1 (1.8)	**52.2** (1.9)	**70.3** (2.1)	72.3 (3.0)	69.2 (1.6)
Other chronic arthritis	155	**65.0** (1.9)	**54.7** (3.4)	**57.3** (2.1)	53.3 (1.8)	**54.5** (1.8)	**69.9** (2.0)	74.1 (2.8)	**70.7** (1.6)
Tendinitis and capsulitis	587	**75.3** (0.8)	**62.9** (1.5)	**66.2** (0.9)	63.1 (0.8)	**60.5** (0.8)	**79.4** (0.9)	83.4 (1.3)	**73.8** (0.7)
No pain condition listed above	1888	**87.8** (0.5)	**85.8** (0.8)	**84.1** (0.5)	**72.8** (0.4)	**69.3** (0.5)	**87.6** (0.5)	89.8 (0.8)	**79.7** (0.4)

SF-36, short-form health survey (36 item): PF, physical functioning; RP, role functioning difficulties caused by physical problems; BP, bodily pain; GH, general health; VT, vitality; SF, social functioning; RE, role functioning difficulties caused by emotional problems; MH, mental health. Higher scores indicate better health status. Pain conditions: FM, fibromyalgia; OA, osteoarthritis; RA, rheumatoid arthritis.

Bold font indicates a significant (p < 0.05) difference between the FM group and the other pain condition group.

### Section 2: cross-sectional clinical studies

Fibromyalgia study participants recruited from outpatient medical centres had significantly lower scores than healthy controls on all eight SF-36 health status domains and the two SF-12 physical and mental summary scales (Table 3). FM patient groups also had a poorer overall health status compared to patients with other specific pain conditions, including myofacial pain syndrome (MPS), SLE, CWP, RA and primary Sjögren's syndrome (prim SS) [Table 3 and data not shown (18,32)]. FM groups scored significantly lower than other specific patient groups on physical functioning [compared with SLE (16), CWP (18), prim SS (32)]; role functioning difficulties caused by physical problems [compared with SLE (16), CWP (18), RA (33), prim SS (32)]; bodily pain [compared with MPS (34), SLE (16), CWP (18), RA (32,33) and prim SS (32)]; general health [compared with MPS (34), CWP (18), RA (32)]; vitality [compared with MPS (34), SLE (16), CWP (18), RA (32,33) and prim SS (32)]; social functioning [compared with CWP (18) and RA (32,33)]; role difficulties caused by emotional problems [compared with MPS (34), SLE (16) and RA (32,33)] and mental health [compared with SLE (16) and RA (32,33)]. Examination of health status summary scores showed that FM patients had a significantly poorer physical health status and a similar mental health status compared with SLE patients; the opposite pattern was observed when comparing summary scores for patients with FM and RA (Table 3).

**Table 3:** SF-36 and SF-12 scores of FM groups and comparison groups in cross-sectional clinical studies

			SF-36 health survey scores (SD)								SF-36 summary scores	
				
Reference (country) Study groups	n*	Mean age (SD)	PF	RP	BP	GH	VT	SF	RE	MH	PCS	MCS
**Pagano et al. (2004) (Brazil)** **(45)**												
FM	40	49.4 (6.0)	33.0^a^ (19.6)	9.38^a^ (22.4)	30.2^a^ (14.83)	45.7^a^ (21.7)	33.1^a^ (21.3)	44.0^a^ (26.5)	30.8^a^ (38.8)	45.6^a^ (22.1)		
Controls	40	49.5 (7.7)	86.6 (16.5)	90.0 (22.5)	76.7 (20.8)	81.5 (17.8)	67.9 (20.4)	82.9 (22.1)	77.5 (37.3)	75.7 (14.8)		
**Ulaş et al. (2006)** **(46)/Alanoğlu et al. (2004)** **(47) (Turkey)**												
FM	34	37 (10)	41.0^a^ (18.0)	13.0^a^ (17.0)	27.0^a^ (15.0)	28.0^a^ (11.0)	26.0^a^ (13.0)	42.0^a^ (21.0)	18.0^a^ (24.0)	36.0^a^ (14.0)		
Controls	22	37 (10)	76.0 (20.0)	93.0 (20.0)	63.0 (18.0)	59.0 (10.0)	68.0 (15.0)	82.0 (19.0)	95.0 (12.0)	66.0 (10.0)		
**Martinez et al. (2001) (Brazil)** **(48)**												
FM	32	36.8	14.8^a^	39.4^a^	26.5^a^	43.3^a^	38.6^b^	45.1^a^	32.3^b^	44.3^b^		
Controls	28	40.5	82.1	84.6	68.9	73.3	59.6	78.0	78.5	67.4		
**Raj et al. (2000) (Canada)** **(49)**												
FM	17	41.8 (6.5)	44.1^a^ (22.0)	10.3^a^ (25.1)	32.9^a^ (15.5)	36.0^a^ (15.6)	25.3^a^ (12.9)	39.7^a^ (18.9)	51.0^b^ (45.8)	57.9^a^ (12.7)		
Controls	14	35.1 (7.7)	96.8 (7.9)	91.1 (21.0)	93.2 (9.1)	71.4 (11.3)	80.7 (7.0)	95.5 (9.3)	95.2 (12.1)	89.7 (11.3)		
**Tüzün (2002) (Turkey)** **(34)**												
FM	33	41.6 (7.3)	67.3^a^ (18.6)	39.1^a^ (37.3)	33.2^a^^e^ (17.7)	36.4^a^^d^ (22.3)	39.3^a^^e^ (19.8)	59.1^b^ (28.2)	44.4^b^^f^ (43.8)	54.9^c^ (21.9)		
MPS	33	40.4 (9.6)	75.2^a^ (15.0)	58.6^b^ (39.8)	45.6^a^ (16.6)	60.7 (23.0)	59.2 (23.0)	71.8 (22.6)	67.6 (37.8)	66.1 (20.7)		
Controls	33	39.0 (5.4)	89.4 (16.4)	83.3 (28.4)	73.6 (22.3)	72.1 (17.7)	60.9 (22.6)	77.7 (25.9)	72.7 (36.8)	66.3 (17.4)		
**DaCosta et al. (2000) (Canada)** **(16)**												
FM	46	46.13 (9.4)	46.7^d^ (26.9)	16.3^d^ (30.8)	32.7^d^ (22.3)	39.1 (26.2)	29.9^d^ (19.09)	53.5^f^ (27.5)	52.9^f^ (41.3)	59.2 (20.8)	29.6^d^ (9.0)	43.12 (10.8)
SLE	59	42.36 (11.3)	65.3 (25.0)	42.4 (43.1)	52.8 (25.5)	44.5 (20.1)	44.1 (19.2)	64.6 (27.5)	70.1 (42.3)	65.0 (19.2)	37.6 (11.1)	46.14 (11.5)
**Neumann et al. (2000) (Israel)** **(18)**												
FM	90	48 (10)	34^b^ (22)	18^b^ (29)	26^b^ (17)	38^b^ (19)	42^b^ (20)	45^b^ (29)	59^b^ (48)	51^c^ (17)		
Newly D_x_ FM	37	58 (11)	28^b^ (23)	14^b^ (29)	17^b^ (16)	20^b^ (16)	40^b^ (21)	22^b^ (20)	49^b^ (46)	48^a^^c^ (18)		
CWP	49	55 (13)	55 (25)	46 (45)	33 (22)	42 (22)	57 (20)	42 (23)	61 (47)	60 (16)		
Controls	50	38 (10)	88 (7)	96 (5)	80 (14)	72 (21)	69 (13)	93 (12)	86 (23)	70 (9)		
**Walker et al. (1997) (USA)**†**(33)**												
FM	36		33.5 (22.7)	8.8^d^ (16.1)	37.5^e^ (16.1)	54.4 (12.1)	22.9^d^ (16.7)	36.7^d^ (22.7)	34.3^d^ (38.9)	56.2^d^ (19.9)	28.0 (6.6)	38.6^d^ (10.3)
RA	33		42.7 (27.1)	32.0 (37.1)	50.4 (21.1)	61.5 (18.0)	46.9 (23.4)	70.7 (28.7)	71.9 (40.4)	75.4 (17.0)	31.7 (9.8)	52.8 (10.5)
**Reisine et al. (2004) (USA)** **(50)**											**SF-12 summary scores**	
FM	287	47.0 (10.8)									29.2^a^ (8.5)	42.1^a^ (11.9)
Controls	286	47.0 (14.0)									49.5 (9.2)	49.8 (10.3)

SF-36, short-form health survey (36 items): SF-12, short-form health survey (12 items); PF, physical functioning; RP, role functioning difficulties caused by physical problems; BP, bodily pain; GH, general health; VT, vitality; SF, social functioning; RE, role functioning difficulties caused by emotional problems; MH, mental health; PCS, physical component summary; MCS, mental component summary. Higher scores indicate better health status. Pain conditions: FM, fibromyalgia; D_x_, diagnosed; CWP, widespread pain; MPS, myofacial pain syndrome; SLE, systemic lupus erythematosus; RA, rheumatoid arthritis.

* All patients in all studies were women.

† Statistical comparisons of FM groups with controls and other diagnostic groups:

a FM group differed significantly from control group, p < 0.001

b FM group differed significantly from control group, p < 0.01

c FM group differed significantly from control group, p < 0.05

d FM group differed significantly from diagnostic group, p < 0.001

e FM group differed significantly from diagnostic group, p < 0.01

f FM group differed significantly from diagnostic group, p < 0.05.

Only one study in the current review found a better health status for the FM group than for the comparison pain group. FM patients had a significantly better health status scores than patients with low back pain (LBP) on the domains of bodily pain, general health, social functioning, role functioning caused by emotional problems and mental health (35). This study was limited by a very small sample size (*n* = 14 and *n* = 10 for FM and LBP respectively). Additional studies are required to examine the differential health status burden of these conditions.

Fibromyalgia was found to be a common and debilitating condition among patients referred to rheumatology because of pain. Of 86 patients with CWP referred to rheumatology, 37 (43%) were found to have previously undiagnosed FM (18). In addition, FM was identified as the most common diagnosis in a group of 145 US Persian War veterans who were referred for rheumatology consultation for medically unexplained symptoms (*n* = 49; 34%) (36). Of the 49 patients with FM, 38 (76%) were males (36). Both studies found FM groups to have significantly poorer health status scores than those without FM (18,36). Findings highlight that FM negatively affects both males and females.

Several studies focussed exclusively on the health status of female patients with FM. However, one study compared health status profiles of male and female patients with FM. Although both males and females with FM had poor health status scores, male FM patients had significantly lower scores than female FM patients on the domain assessing role functioning difficulties caused by emotional problems (Table 4). Although FM is less common in males than in females, males with FM may be at even greater risk for experiencing reduced health status than their female counterparts.

**Table 4:** SF-36 scores in male and female FM patients

			SF-36 health survey scores (SD)							
			
Reference (country) Study groups	n	Mean age (SD)	PF	RP	BP	GH	VT	SF	RE	MH
**Buskila et al. (2000)** **(51) (Israel)**										
FM males	40	45.0 (13.0)	40.6 (24.1)	8.3 (18.4)	24.9 (17.0)	26.9 (14.6)	36.3 (19.7)	32.4 (26.4)	23.9^a^ (41.8)	46.5 (17.5)
FM females	40	46.0 (10.0)	33.6 (25.1)	11.3 (23.3)	27.9 (14.5)	32.6 (13.7)	39.8 (12.3)	37.2 (21.4)	60.8 (38.4)	50.4 (10.6)

SF-36, short-form health survey (36 items): PF, physical functioning; RP, role functioning difficulties caused by physical problems; BP, bodily pain; GH, general health; VT, vitality; SF, social functioning; RE, role functioning difficulties caused by emotional problems; MH, mental health. Higher scores indicate better health status.

a FM males differed significantly from FM females, p < 0.001.

### Section 3: FM clinical trials

A total of 14 FM clinical trials included assessment of health status using the SF-36 or the SF-12 (Table 5). These trials examined the impact of a variety of pharmacological and non-pharmacological treatments for FM. Before receiving a new treatment, trials participants had poor health status scores (Table 5). All of the trials reported a significant improvement from baseline on at least one aspect of health status. Without exception, findings from placebo-controlled trials (37–39) showed significant improvements favouring an active treatment vs. placebo on specific physical and mental health status domains. It is beyond the score of this review to describe the magnitude of treatment effects associated with the different interventions. However, findings suggest that effective treatment can lead to significant improvements in aspects of both physical and mental health status.

**Table 5:** Baseline SF-36 or SF-12 scores of patients in clinical trials of FM

			Mean score on health status domains (SD)†								Summary scores	
				
Health status measure used Reference (country)	*n* (% women)	Mean age (SD)†	PF	RP	BP	GH	VT	SF	RE	MH	PSC	MCS
**SF-36**												
Arnold et al. (2004) (38) (USA)	207 (89)	49.1	45.5	17.0	31.9	50.4	23.8	58.3	59.1	63.7	29.8	45.2
Assis et al. (2006) (52) (Brazil)	60 (100)	42.8									64.9	57.2
Bennett et al. (2003) (37)/Bennett et al.(2005) (9) (USA)	315 (94)	50	38.5	11.5	28	46.5	20	49.5	42	59.5	28.5	41.5
Crofford et al. (2005) (39) (USA)	529 (90)	47.6	40.8	15.4	27.8	47.7	20.5	49.2	46.4	58.9		
Donaldson et al. (2001) (USA) (53)	26 (93)	NR	36.3 (24.3)	6.3 (11.1)	32.6 (20.2)	44.1 (21.6)	18.0 (14.4)	40.0 (26.5)	25.0 (26.5)	57.2 (23.1)		
Faull (2005) (13) (New Zealand)	13 (100)	46.3 (12.3)	51.7	44.6	39.3	48.5	34.6	64.4	64.4	66.6		
Fregni et al. (2006) (54) (Brazil)	32 (100)	53.0	30.9	33.6	37.9	43.6	41.7	42.3	33.6	39.2		
Mannerkorpi et al. (2000) (55) (Sweden)	26 (100)	45.0 (8.0)	44.3 (20.0)	16.1 (29.0)	24.7 (17.4)	38.3 (16.8)	22.5 (14.5)	46.9 (26.9)	47.6 (43.9)	59.9 (20.4)		
Neumann et al. (2001) (56) (Israel)	48 (100)	54.45	34	19	19	27	39	42	48	51		
Valim et al. (2003) (57) (Brazil)	76 (100)	46.8	55.0	23.1	34.9	49.8	31.4	52.2	39.8	43.0	36.3	35.7
Vitorino et al. (2006) (58) (Brazil)	50 (100)	47.7 (8.8)	35.5	16.7	19.7	34.6	29.6	38.5	15.0	40.4		
Zijlstra et al. (2005) (59) (the Netherlands)											28.2	46.1
**SF-36 comparison treatment group studies**‡**(15)**												
FM (USA)	18 (94)	51.2 (11.2)	61.7 (24.0)	38.9 (37.6)	39.2 (20.8)	60.1 (22.4)	32.8 (22.4)	61.1 (24.2)	53.7 (44.5)	68.2 (15.9)		
Incontinence (USA)	30 (90)	76.9 (8.1)	11.1 (18.7)	21.7 (35.2)	46.6 (31.3)	42.6 (20.7)	40.7 (21.5)	62.5 (32.3)	42.2 (45.4)	69.6 (21.1)		
Prostate cancer (USA)	12 (0)	63.4 (9.7)	93.3 (20.0)	91.7 (28.9)	80.8 (23.7)	69.8 (21.8)	73.8 (25.6)	85.4 (26.6)	97.2 (9.6)	79.3 (16.2)		
COPD (USA)	13 (62)	66.7 (3.7)	23.1 (15.2)	9.6 (21.7)	58.5 (24.2)	35.5 (15.3)	40.8 (14.1)	52.9 (18.5)	41.0 (45.5)	59.1 (15.3)		
AIDS (USA)	35 (14)	39.5 (6.0)	47.4 (33.3)	87.9 (31.1)	40.5 (33.2)	30.1 (22.9)	35.3 (23.6)	52.5 (34.1)	41.9 (44.5)	69.6 (22.8)		
Hyperlipidaemia (USA)	213 (47)	46.1 (9.0)	89.6 (15.2)	80.8 (33.1)	73.1 (22.3)	76.5 (16.0)	61.4 (19.8)	87.7 (18.5)	80.3 (30.2)	75.6 (16.6)		
**SF-12**												
Hidalgo et al. (2006) (60) (Spain)	35 (93)	47.2 (7.9)									27.7 (7.7)	29.5 (37.5)

SF-36, short-form health survey (36 items): SF-12, short-form health survey (12 items); PF, physical functioning; RP, role functioning difficulties caused by physical problems; BP, bodily pain; GH, general health; VT, vitality; SF, social functioning; RE, role functioning difficulties caused by emotional problems; MH, mental health; PCS, physical component summary; MCS, mental component summary. Higher scores indicate better health status. Pain conditions: FM, fibromyalgia; COPD, chronic obstructive pulmonary disease; AIDS, acquired immune deficiency syndrome.

† SD is not shown when mean values were calculated by averaging baseline scores across treatment groups.

‡ Schlenk et al. (1998) (15) reported on baseline data of subjects from six treatment studies of six chronic disorders.

### Section 4: health status profiles of FM groups with and without a concurrent pain condition

**Figure 1:**
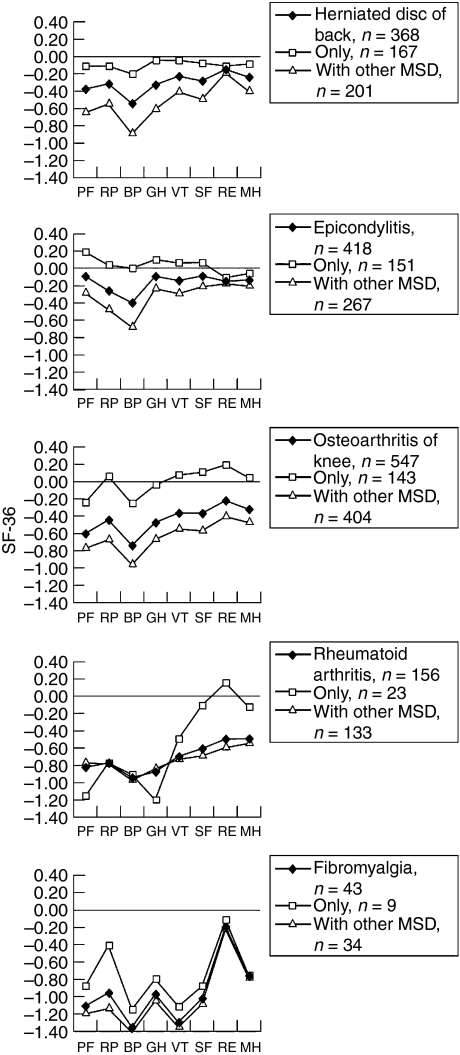
Patterns of health status for pain conditions compared with the general population. SF-36 scores expressed as number of standard deviations from the population mean. PF, physical functioning; RP, role functioning difficulties caused by physical problems; BP, bodily pain; GH, general health; VT, vitality; SF, social functioning; RE, role functioning difficulties caused by emotional problems; MH, mental health. Picavet HSJ, Hoeymans N. Health related quality of life in multiple musculoskeletal diseases: SF-36 and EQ-5D in the DMC3 study. Annals of the Rheumatic Diseases 2004; 63; 723–729. Adapted with permission from the BMJ Publishing Group

People with a specific pain condition commonly had at least one concurrent (i.e. coexisting) pain condition (Table 6; Figure 1). Health status deteriorated in conjunction with the number of pain conditions present (Table 6; Figure 1). This pattern was observed for five specific pain conditions, including FM (Figure 1). Importantly, however, FM remained significantly impairing even when it occurred alone. Respondents with FM alone had standardised scores that were 1 SD below the general population in the areas of bodily pain and vitality (Figure 1). This pattern of findings was unique to FM (Figure 1). Findings highlight that impaired bodily pain and decreased vitality are core features of FM. Future studies with larger samples without concurrent health conditions are required to confirm findings.

**Table 6:** SF-36 scores of people in the general population, by number of pain conditions

		SF-36 health survey scores (SD)							
		
Reference (country) Study groups	n	PF	RP	BP	GH	VT	SF	RE	MH
**Picavet et al. (2003)(14, p.725)(the Netherlands)**	3664								
1 pain condition*	957	80.0 (0.6)	74.3 (1.2)	73.8 (0.7)	67.7 (0.6)	64.6 (0.6)	83.2 (0.7)	86.7 (1.0)	76.0 (0.6)
2 pain conditions*	478	72.7 (1.0)	63.0 (1.8)	65.5 (1.0)	64.0 (0.9)	60.2 (1.0)	79.6 (1.1)	84.0 (1.5)	73.8 (0.8)
3 pain conditions*	193	63.4 (1.6)	53.2 (3.0)	57.0 (1.8)	55.8 (1.6)	56.0 (1.6)	69.1 (1.8)	76.0 (2.6)	69.9 (1.4)
No pain condition*	1888	87.8 (0.5)	85.8 (0.8)	84.1 (0.5)	72.8 (0.4)	69.3 (0.5)	87.6 (0.5)	89.8 (0.8)	79.9 (0.4)
Any pain condition*	1776	75.2 (0.5)	67.1 (0.9)	68.5 (0.6)	64.6 (0.5)	61.6 (0.5)	79.8 (0.6)	83.7 (0.8)	74.3 (0.4)

SF-36, short-form health survey (36 items); PF, physical functioning; RP, role functioning difficulties caused by physical problems; BP, bodily pain; GH, general health; VT, vitality; SF, social functioning; RE, role functioning difficulties caused by emotional problems; MH, mental health; PCS, physical component summary; MCS, mental component summary. Higher scores indicate better health status.

* Table 2 shows specific pain conditions.

Several studies examined the extent to which FM contributed to the overall health status burden of patients with and without a specific concurrent condition (Table 7). The presence of FM added to the health status impairment among patients who also had migraine, RA or SLE (Table 7). In contrast, headache did not significantly add to the health status burden of patients with FM (Table 7). Findings highlight that FM uniquely contributes to the overall health status burden in affected persons.

**Table 7:** SF-36 scores in patients with and without a specific concurrent condition

			SF-36 health survey scores (SD)								SF-36 summary scores	
				
Reference (country) Study groups	*n* (% women)	Mean age (SD)	PF	RP	BP	GH	VT	SF	RE	MH	PCS	MCS
**Ifergane et al. (2006)(40) (Israel)**	(72)											
Migraine + FM	16	43.4 (11.4)	58.4^a^ (17.1)	39.1^c^ (40.8)	38.4 (11.8)	40.9^a^ (22.4)	35.3^b^ (18.9)	57.6 (13.1)	68.8 (41.2)	59.5 (18.7)		
Migraine no FM	56	43.6 (12.5)	85.4 (18.3)	63.4 (40.2)	42.3 (11.4)	70.1 (23.3)	49.4 (18.8)	50.4 (8.6)	75.6 (39.4)	67.8 (19.1)		
**Alarcon et al. (2004)(41) (USA)**	346	37.7 (13.0)										
SLE + FM	17	NR	30.5^a^	8.3^a^	20.1^a^	30.4^c^	15.6^a^	36.7^a^	31.1^c^	49.7^c^	24.5^a^	36.7^c^
SLE no FM	329	NR	55.1	44.8	52.6	44.0	43.8	63.9	58.2	66.9	36.4	45.9
Wolfe & Michaud (2004)*(12) (USA)	11866											
RA + FM	2078 (84)	54.5 (14.2)									23.5 (6.6)	29.5 (12.8)
RA no FM	9788 (76)	47.4 (12.9)									33.5 (10.2)	46.1 (12.7)
**Marcus et al. (2005)(43) (USA)**	100 (96)	47.6 (11.9)										
FM no headache			33.1 (4.3)	6.3 (3.5)	20.8 (2.6)	34.4 (4.4)	20.2 (4.0)	43.2 (4.7)	15.3 (7.7)	56.8 (4.1)		
FM + headache			34.9 ns (2.4)	7.9 ns (2.0)	22.5 ns (1.5)	34.9 ns (2.5)	22.6 ns (2.2)	38.0 ns (2.6)	35.5 ns (4.4)	51.5 ns (2.3)		

SF-36, short-form health survey (36 items): SF-12, short-form health survey (12 items); PF, physical functioning; RP, role functioning difficulties caused by physical problems; BP, bodily pain; GH, general health; VT, vitality; SF, social functioning; RE, role functioning difficulties caused by emotional problems; MH, mental health; PCS, physical component summary; MCS, mental component summary. Higher scores indicate better health status. Conditions: FM, fibromyalgia; RA, rheumatoid arthritis; SLE, systemic lupus erythematosus.

* Investigators did not report p-values for group comparisons, as essentially all differences were significant at the 0.05 level because of the very large sample sizes. Investigators interpreted the SF-36 scores of the FM group as ‘very abnormal’.

Statistical comparisons:

a Condition + FM group differed significantly from condition group without FM, p < 0.001

b Condition + FM group differed significantly from condition group without FM, p < 0.01

c Condition + FM group differed significantly from condition group without FM, p < 0.05.

The extent to which FM was associated with mental and physical health status was examined, after adjusting for the presence of non-rheumatic chronic diseases and sociodemographic characteristics (1). Health status was examined using the SF-12. FM was the only pain condition uniquely associated with the mental component of health status (adjusted MCS 30.0, 95% CI 34.6–43.4) but not the physical component of health status (adjusted PCS 33.9, 95% CI 29.3–38.5). The opposite pattern was observed for RA (adjusted PCS 29.1, 95% CI 21.9–36.2; adjusted MCS 42.8, 95% CI 36.4–49.2), OA of the knee (adjusted PCS 31.7, 95% CI 27.3–36.1; adjusted MCS 43.9, 95% CI 39.8–48.0) and LBP (adjusted PCS 32.4, 95% CI 28.0–36.8; adjusted MCS 43.0, 95% 38.8–47.2).

## Discussion

Studies performed around the world showed that people with FM had a remarkably consistent pattern of health status impairment. People with FM scored significantly lower on all eight health status domains compared with people in the general population. FM groups had mental health summary scores that fell 1 SD below the general population mean and physical health summary scores that fell 2 SD below the general population mean. People with FM also had similar or significantly lower scores on all eight health status domains compared to people with other specific pain conditions, including RA, OA, SLE, prim SS and MPS (14,16,32–34). FM groups had significantly lower scores than all of the specific pain conditions described above on domains of bodily pain and vitality (14,16,32–34).

To provide a broader interpretive context for understanding the health status burden of FM, SF-36 scale scores of FM patient groups can be compared with those of norms for MOS patients (Table 3 and Table 1). Without exception, FM patient groups had numerically lower scores on all eight health status domains compared with norms for MOS patients with hypertension, recent acute MI and type II diabetes. Similar findings were observed when FM scores were compared with norms for MOS patients with congestive heart failure and chronic obstructive pulmonary disease, with an occasional exception where a numerically higher score was found for a FM group on a physical status domain. Compared with norms for MOS patients with depression, FM groups consistently had numerically lower scores on physical domains, but not on mental domains assessing role difficulties caused by emotional problems and mental health. These are the only two SF-36 domains that exclusively measure mental aspects of health status. Although based on numerical comparisons as opposed to statistical analysis, these findings suggest that the overall health status burden of FM is at least as great in magnitude as that of a variety of health conditions widely accepted as impairing.

Studies in this review primarily reported on the health status of women. Two studies, however, showed that FM also reduced the health status of males. A study comparing health status profiles of male and female FM patients showed that males had an even poorer health status than females (18). In addition, FM was the most common diagnosis in 145 US veterans who were referred for rheumatologic evaluation for medically unexplained symptoms (36). Most (76%) of these FM patients were male. Findings highlight that FM also imposes a significant health status burden on males.

Health status impairments of people with FM could not be fully explained by the presence of other concurrent health conditions. People with FM (both with and without another concurrent pain condition) had standardised scores that were at least 1 SD below the general population mean on domains of bodily pain and vitality (14). This pattern of findings was unique to people with FM. Findings highlight that impairments in bodily pain and vitality are central features of FM. Moreover, FM significantly added to physical and mental health status impairments in patients who also had migraine, SLE or RA (12,40–42). In contrast, headache did not add significantly to the overall health status burden in FM patients (43). Together, findings underscore that FM makes a unique contribution to the health status burden of people with the condition.

This review is subject to several limitations. First, we only considered studies that measured health status with the SF-36 and the SF-12. However, these generic instruments permit comparisons across groups with and without FM while disease-specific instruments do not. Second, all health status data were based on self-report. However, as no objective clinical markers exist for FM in routine clinical practice, clinical decisions depend on FM patients’ self-reported symptoms, treatment side effects and their combined impact on health status. The centrality of the patient's point of view is also emphasised in clinical research (10,44). Third, many of the studies in this review had small sample sizes. However, even with the small sample sizes, significant differences were observed between groups with and without FM. These studies also revealed a consistent pattern of health status impairment among people with FM.

Although FM is a controversial construct, studies performed around the world showed that people with FM have substantial impairments in both physical and mental health status. People with FM had a health status burden that was greater in magnitude compared to those with health conditions that are widely accepted as impairing. Findings from FM clinical trials suggest that efficacious treatments can improve aspects of health status, although findings require confirmation in usual care settings. Findings in this review underscore the importance of addressing the substantial health status burden of people with FM, irrespective of current debate about how FM should be classified.
